# A novel intelligent system based on adjustable classifier models for diagnosing heart sounds

**DOI:** 10.1038/s41598-021-04136-4

**Published:** 2022-01-25

**Authors:** Shuping Sun, Tingting Huang, Biqiang Zhang, Peiguang He, Long Yan, Dongdong Fan, Jiale Zhang, Jinbo Chen

**Affiliations:** 1 School of Information Science and Engineering, Hunan Institute of Science and Technology, 414006 Yueyang, China; 2 Department of Information Engineering, Nanyang Institute of Technology, 473004 Nanyang, China

**Keywords:** Cardiology, Health care, Engineering, Mathematics and computing

## Abstract

A novel intelligent diagnostic system is proposed to diagnose heart sounds (**HS**s). The innovations of this system are primarily reflected in the automatic segmentation and extraction of the first complex sound $({ CS }_{1})$ and second complex sound $({ CS }_{2})$; the automatic extraction of the secondary envelope-based diagnostic features $\gamma _{_1}$, $\gamma _{_2}$, and $\gamma _{_3}$ from ${ CS }_{1}$ and ${ CS }_{2}$; and the adjustable classifier models that correspond to the confidence bounds of the Chi-square ($\chi ^{2}$) distribution and are adjusted by the given confidence levels (denoted as $\beta$). The three stages of the proposed system are summarized as follows. In stage 1, the short time modified Hilbert transform (**STMHT**)-based curve is used to segment and extract ${ CS }_{1}$ and ${ CS }_{2}$. In stage 2, the envelopes ${ CS _{1}}_{\mathrm{F_{E}}}$ and ${ CS _{2}}_{\mathrm{F_{E}}}$ for periods ${ CS }_{1}$ and ${ CS }_{2}$ are obtained via a novel method, and the frequency features are automatically extracted from ${ CS _{1}}_{\mathrm{F_{E}}}$ and ${ CS _{2}}_{\mathrm{F_{E}}}$ by setting different threshold value ($Thv$) lines. Finally, the first three principal components determined based on principal component analysis (**PCA**) are used as the diagnostic features. In stage 3, a Gaussian mixture model (**GMM**)-based component objective function $f_{ et }(\mathbf{x })$ is generated. Then, the $\chi ^{2}$ distribution for component *k* is determined by calculating the Mahalanobis distance from ${\mathbf{x }}$ to the class mean $\mu _{_k}$ for component *k*, and the confidence region of component *k* is determined by adjusting the optimal confidence level $\beta _{k}$ and used as the criterion to diagnose **HS**s. The performance evaluation was validated by sounds from online **HS** databases and clinical heart databases. The accuracy of the proposed method was compared to the accuracies of other state-of-the-art methods, and the highest classification accuracies of $99.43\%$, $98.93\%$, $99.13\%$, $99.85\%$, $98.62\%$, 99.67$\%$ and 99.91$\%$ in the detection of **MR**, **MS**, **ASD**, **NM**, **AS**, **AR** and **VSD** sounds were achieved by setting $\beta _{k}(k=1, 2, \ldots , 7)$ to 0.87,0.65,0.67,0.65,0.67,0.79 and 0.87, respectively.

## Introduction

### Background

As an efficient method, using heart sound (**HS**) analysis is often used to evaluate heart function; this approach has been widely used to diagnose heart disease and evaluate heart functions, such as congenital heart disease classification^1^, ventricular septal defect detection^2^, blood pressure estimation^3^ and congenital heart disease screening^4^, for children and adults. A normal **HS** is primarily composed of two basic sounds: the first sound (${ S }_{1}$) which is generated by the closing of aortic valves and the vibrations associated with tensing of the chordate trendiness and the ventricular walls, the second sound (${ S }_{2}$) is produced by the closure of the aortic and pulmonic valves at the beginning of is volumetric ventricular relaxation. However, **HS**s with unitary murmurs generally occur between ${ S }_{1}$ and ${ S }_{2}$ with different noise patterns^5^. Therefore, analyses of ${ S }_{1}$, ${ S 2}$, and the period between ${ S }_{1}$ and ${ S }_{2}$ play important roles in characterizing **HS** features with different types of information. Detailed information for ${ S }_{1}$, ${ S }_{2}$, and the sounds between ${ S }_{1}$ and ${ S }_{2}$ can be used to accurately classify **HS**. Additionally, to avoid analyzing the sounds between ${ S }_{1}$ and ${ S }_{2}$, which are generally segmented from **HS**s with low accuracy, ${ S }_{1}$ and part of the period between ${ S }_{1}$ and ${ S }_{2}$ are integrated to obtain ${ CS }_{1}$, and ${ S }_{2}$ and the part of the period between $S _{1}$ and $S _{2}$ are integrated to form ${ CS }_{2}$. Then, the features are efficiently extracted from ${ CS }_{1}$ and ${ CS _{2}}$. Finally, a classification method is established to diagnose heart diseases.

### Need for research

- $\bullet$ ${\varvec{CS}}_{1}$ **and** ${\varvec{CS}}_{2}$ **extraction** The studies regarding **HS** segmentation can be summarized into two branches: one branch includes studies that segment each cardiac cycle into a sequence of four heart stages: ${ S }_{1}\longrightarrow$ *Systole period* $\longrightarrow { S }_{2}\longrightarrow$ *Diastole period*^6,7^. As a result, the four fundamental stages to be segmented are different due to the nonstationary nature of an abnormal **HS**s signal and the effect of background noise. The other branch includes studies that segment a periodic **HS**s into a sequence of two heart stages, which are expressed as $$\mathop{{ CS }_{1}}\limits _{\begin{array}{c} {\overbrace{{{One part of Diastole}} \rightarrow {{ S }_{1}} \rightarrow {{One part of Systole}}}} \end{array}}$$ $\rightarrow$ $$\mathop{{ CS }_{2}}\limits _{\begin{array}{c} {\overbrace{{{The other of Diastole}} \rightarrow {{ S }_{2}} \rightarrow {{The other of Systole}}}} \end{array}}$$ based on the **STMHT** algorithm; this approach was reported to be successfully applied in diagnosing heart diseases, such as in ventricular septal defect (**VSD**) diagnosis^8^ and several kinds of heart disease diagnosis^9^. Moreover, study^9^ noted that the use of frequency features was more efficient in distinguishing normal from abnormal sounds than was the use of time features. Therefore, an efficient frequency feature extraction method should be developed.
- $\bullet$ **Feature extraction** As an important component of efficient feature extraction, the frequency width of the envelope over a given threshold value ($Thv$) has been verified to be useful for detecting heart diseases^8–11^. However, for many types of **HS**s, it is difficult to extract frequency widths with an unsuitable $Thv$ due to the existence of a non smooth envelope. To extract the frequency widths for a smooth envelope without setting different $Thv$ values, the smooth envelope can be treated as a secondary envelope, as proposed in^9^, and used to automatically extract the frequency feature matrix based on the **STMHT** technique; this method was successfully applied to detect different types of heart diseases. However, for mitral stenosis and mitral regurgitation noises, the feature matrix was not easily extracted because the second frequency component was missing. Therefore, to improve the classification accuracy for diagnosing different types of heart disease and simplify the complexity of the diagnostic method, the smooth envelopes for ${ CS }_{1}$ and ${ CS }_{2}$ extraction in the frequency domain must be considered; additionally, more frequency widths corresponding to different $Thv$ values should be used, and dimensionality reduction should be employed to reduce the number of features considered . Such a classification method could be applied in the efficient extraction of features for diagnosing heart diseases.
- $\bullet$ **Classifier model** Gaussian mixture models (**GMM**s) have been used in a wide variety of clustering applications^12–18^ due to their powerful mathematical characteristics. Confidence regions are used to diagnose the detection data **x** in **GMM**s, and the optimal confidence regions is determined based on Mahalanobis distance following the Chi-square ($\chi ^{2}$) distribution. Thus, classifier models with adjustable sizes corresponding to the confidence bounds of the Chi-square ($\chi ^{2}$) distribution, which can be adjusted by changing the desired confidence level (denoted as $\beta$), are proposed. The $\chi ^{2}$ confidence bounds used as the classification criteria are employed to diagnose heart diseases.

### Major contributions and organization

In summary, this study proposes an innovative and intelligent system. The major contributions in this study are (1) the **STMHT**-based ${ CS }_{1}$ and ${ CS }_{2}$ are automatically located and extracted; (2) a novel method for obtaining the secondary curves of ${ CS }_{1}$ and ${{ CS }_{2}}$ are extracted in the frequency domain; (3) frequency features are automatically extracted over the given threshold value; (4) the diagnostic features $\gamma _{_1}$, $\gamma _{_{2}}$ and $\gamma _{_3}$ are determined based on **PCA**; and (5) the confidence region of the $\chi ^{2}$ distribution, which are adjusted based on the desired $\beta$, is determined and used as the classification criterion for diagnosing a given **HS**. The remainder of this paper is organized as follows. Section “Methodology” presents the approach for determining the diagnostic features $[\gamma _{_1}, \gamma _{_2}, \gamma _{_3}]$, and a definition of the confidence region-based diagnostic method for diagnosing heart diseases. In “Performance evaluation” section, the performance of the proposed method is compared with that of other efficient methods for diagnosing heart diseases. In “Conclusion” section, the conclusions are provided. Finally, the future study is pointed out in “Future study”.

## Methodology

This study was approved by the ethics committee of Nanyang Institute of Technology (Approval Number:2016-06) and the informed consent was waived by the ethics committee of Nanyang Institute of Technology. The present study was also conducted in accordance with the tenets of the 1975 Declaration of Helsinki, as revised in 2008^19^.

**Figure 1:**
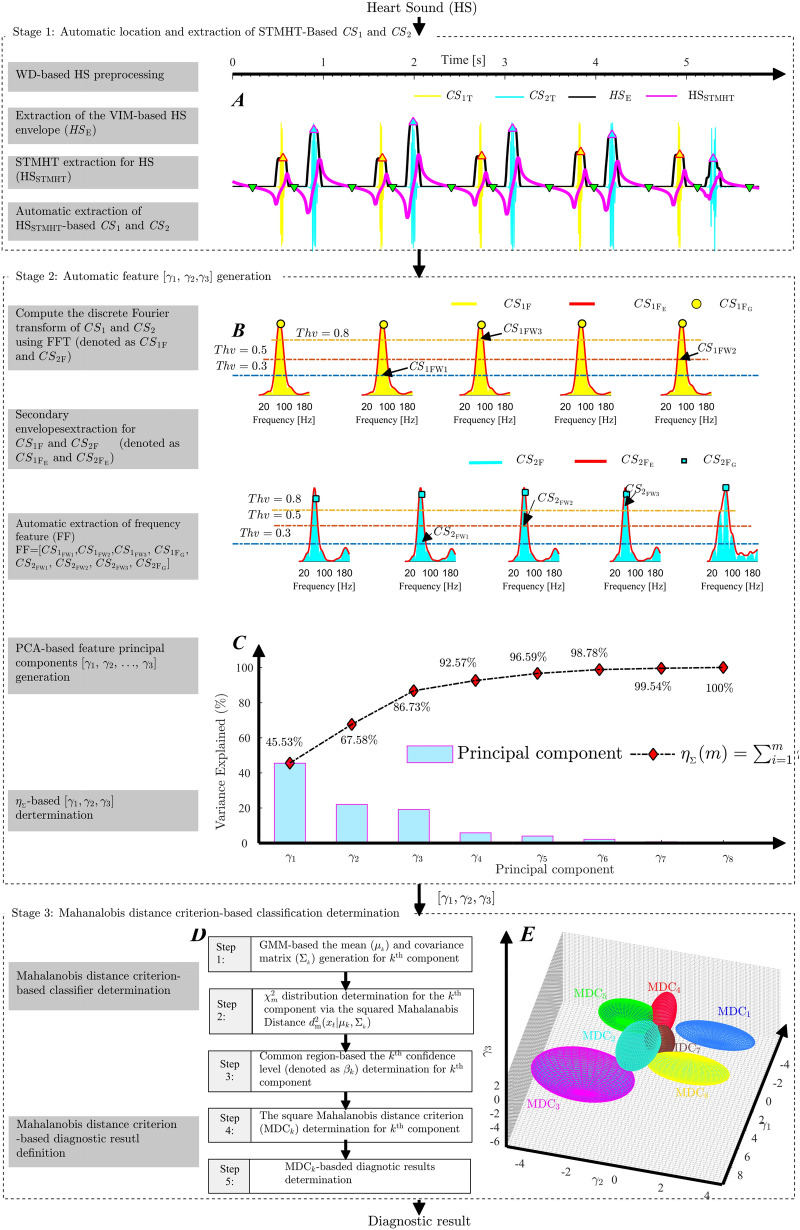
Flow chart of the proposed methodology.

The flow chart of the proposed intelligent system, shown in Fig. 1, consists of three stages: the automatic location and extraction of ${ CS }_{1}$ and ${ CS }_{2}$; the automatic determination of frequency features $\gamma _{1}$, $\gamma _{2}$ and $\gamma _{3}$; and the establishment of the Mahalanobis distance criterion-based diagnostic method. In stage 1, the **STMHT**-based curve (denoted as ${\mathrm{HS}}_{\mathrm{STMHT}}$), which is extracted for the ${\mathrm{HS}}_{\mathrm{E}}$ envelope generated by the **HS**, is used to segment and extract ${ CS }_{1}$ and ${ CS }_{2}$ from the **HS** (Fig. 1A). In stage 2, the envelopes ${ CS 1}_{\mathrm{F_{E}}}$ and ${ CS 2}_{\mathrm{F_{E}}}$ for every period ${ CS }1$ and ${ CS }2$ are obtained via a novel method, and the frequency features are automatically extracted from ${ CS _{1}}_{\mathrm{F_{E}}}$ and ${ CS _{2}}_{\mathrm{F_{E}}}$ by setting different $Thv$ lines. Finally, the first three principal components, $\gamma _{1}$, $\gamma _{2}$ and $\gamma _{3}$, which express $86.7\%$ of the $\mathrm{FF}$ information, are determined and used as diagnostic features (Fig. 1B, C). In stage 3, the **GMM**-based mixed classification objective function $f_{ et }(\mathbf{x})$ which combines component *k* with respect to the parameters $\pi _{k}$, $\mu _{k}$, and $\Sigma _{k}$ and the features ${\mathbf{x}}=[\gamma _{_1}, \gamma _{_2}, \gamma _{_3}]$, is generated. Then, the $\chi ^{2}$ distribution for component *k* is determined by calculating the Mahalanobis distance from **x** to the class mean $\mu _{_k}$ of component *k*, and the adjustable confidence bound (denoted as ${\mathrm{MDC}}_{k}$ shown in Fig. 1E) is determined to diagnose heart diseases.

### Stage 1: Automatic extraction of $CS _{1}$ and $CS _{2}$

**Figure 2:**
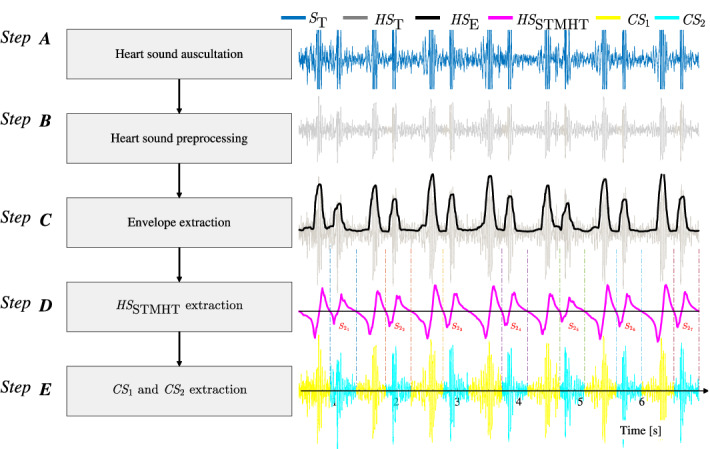
Flow chart of the $CS _1$ and $CS _2$ extraction.

As shown in Fig. 2, five steps consisting of heart sound auscultation, heart sound preprocessing, heart sound envelope extraction, STMHT extraction, and $CS _{1}$ and $CS _{2}$ extraction, which is used to construct the procedure of the $CS _{1}$ and $CS _{2}$ extraction and is detailed in the following steps.

#### Step A: Heart sound auscultation

Auscultation is performed for the purposes of examination cardiovascular. As described in previous study^8^, the original heart sound, denoted as $S_{\mathrm{T}}$ (colored in blue line as shown in Fig. 2), are collected by 3M-3200 electronic stethoscope with a ${F}_{s}=44.1 k$Hz sample rate which is widely used by many doctors and produced by American 3M company^20^, and the tricuspid area is selected as the auscultation area due to the tricuspid area reported to supply more important information^21^. Meanwhile, you can hear the sounds when auscultating heart sounds, ensuring that we avoid as much environmental noise as possible during the auscultation procedure. Even so, the collected heart sounds still need to be preprocessing for canceling the invalid components.

#### Step B: WD-based heart sound preprocessing

**HS**s are reported to be primarily dispersed in the frequency range of 20$\sim$700 Hz^2,8,9^. Therefore, according to the sampling frequency (${\mathrm{Fs}}=44.1$ kHz), WD-based **HS**s are filtered to obtain the efficient frequency components ($21.5 \sim 689$ Hz). The Daubechies wavelet 10 (*d*B10) has been used to give the maximum signal-to-noise ratio and minimum root-mean-square error for **HS**s^22^. Therefore, *d*B10 is selected for use as the mother wavelet for preprocessing **HS**s. A filtered and normalized sound, colored by gray and denoted as ${ HS }_{\mathrm{T}}$, is shown in Fig. 2.

#### Step C: heart sound envelope $({ HS }_{\mathrm{E}})$ extraction

The Viola integral-based envelope, denoted as ${ HS }_{\mathrm{E}}$, is extracted from the heart sound ${ HS }_{\mathrm{T}}$, as reported in studies^8,9^; this envelope can effective overcome amplitude variations and complex backgrounds and noise. This concept is described as follows. Consider a filtered sound ${ HS }_{\mathrm{T}}[m]$ for $m=0,1,\ldots ,M-1$, where *M* denotes the number of **HS**s. In a $W_{\mathrm{m}}$ neighborhood of time *m*, called the width $W_{\mathrm{m}}$ time scale, the *M*-point envelope ${ HS }_{\mathrm{E}}[m]$ is obtained by Eq. (1):

1 $$\begin{aligned} {HS}_{\mathrm{E}}[m]=\frac{1}{2W_{\mathrm{m}}+1}\sum _{k=m-W_{\mathrm{m}}}^{m+W_{\mathrm{m}}}\left( { HS }_{\mathrm{T}}[k]-{\overline{HS}}_{\mathrm{T}}[m]\right) ^2, m=W_{\mathrm{m}}, W_{\mathrm{m}}+1,\ldots M-1-W_{\mathrm{m}}, \end{aligned}$$

where

2 $$\begin{aligned} \overline{ HS }_{\mathrm{T}}[m]=\frac{1}{2W_{\mathrm{m}}+1}\sum _{k=m-W_{\mathrm{m}}}^{m+W_{\mathrm{m}}}{ HS }_{\mathrm{T}}[k], \end{aligned}$$

$W_{\mathrm{m}}=2205$ if the duration of ${ CS }_{1}$ or ${ CS }_{2}$ greater than 0.13 s. Finally, normalization is performed by setting the maximum amplitude of ${ HS }_{\mathrm{E}}$ to 1 (Fig. 2).

#### Step D: **STMHT** extraction for **HS**

Given an *M*-point **HS**, the **STMHT** for the **HS**s , ${ HS }_{\mathrm{STMHT}}$, is computed from Eq. (3)

3 $$\begin{aligned} {\mathrm{HS}}_{\mathrm{STMHT}}[n]= & \sum _{m=n-\frac{N-1}{2}}^{n+\frac{N-1}{2}}{{ HS }_{\mathrm{E}}[m]W_{N}[m-n]W_{\mathrm{E}}\left[m-(n-\frac{N-1}{2})\right]}, \end{aligned}$$

4 $$\begin{aligned} W_{\mathrm{E}}[i]= & {\left\{ \begin{array}{ll} \frac{cos(\frac{N-1-2i}{2N}\pi )-cos(\frac{N-1-2i}{2}\pi )}{Nsin(\frac{N-1-2i}{2N}\pi )}& \text {for}\quad i=0,1,\ldots ,N-1\\ 0 & \text {for} \quad i=\frac{N-1}{2}\\ \end{array}\right. }. \end{aligned}$$

where $n=(N-1)/2,\ldots , M-1-(N-1)/2$, and $W_{N}[l](l=-(N-1)/2,\ldots ,(N-1)/2)$ is a moving window of odd length *N*. According to studies^2,8^, the length *N* is set to 44101.

#### Step E: Automatic extraction of ${ CS }_{1}$ and ${ CS }_{2}$

**Figure 3:**
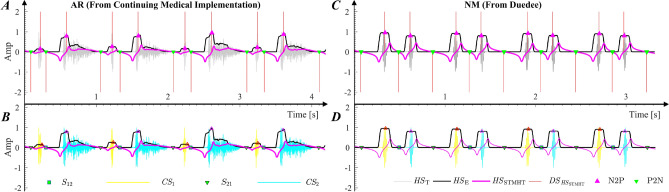
The automatic extraction procedures for ${ CS }_{1}$ and ${ CS }_{2}$. **A-B** show the procedure for an example of a typical **AR** from the database in^26^. **C-D** show the procedure for an example of a typical normal sound database^27^.

The characteristics of ${ HS }_{\mathrm{STMHT}}$ considered in studies^2,8^, as shown in Fig. 3A, C, are summarized as follows: ${\textcircled {1}}$ The negative-to-positive (**N2P**) points of ${ HS }_{\mathrm{STMHT}}$, denoted by ${{\blacktriangle }}$, correspond to the geometry center peaks of $S _{1}$ and $S _{2}$; ${\textcircled {2}}$ The geometry center between ${ S }_{1}$ and ${ S }_{2}$, denoted by ${{\blacktriangledown }}$ is determined by the positive-to-negative **P2N** points of $HS _{\mathrm{STMHT}}$. Moreover, the interval from $S _{2}$ to $S _{1}$ is generally greater than that from $S _{1}$ to $S _{2}$ in one period of an **HS**^23–25^. Therefore, the **N2P** and **P2N**-based $CS _{1}$ and $CS _{2}$ features can be automatically segmented from one period of an **HS** and extracted by two procedures, as described as follows.

**(1)**: $${\mathrm{N2P}}$$ **and** $${\mathrm{P2N}}$$ **location**
  The algorithm for detecting $\mathrm{N2P}$ and $\mathrm{P2N}$ is detailed as follows.

- ${\textcircled {{1}}}$ First, the signum function of ${ HS }_{\mathrm{STMHT}}$, denoted as $S_{{ HS }_{\mathrm{STMHT}}}$, is calculated by
  5 $$\begin{aligned} S_{{ HS }_{\mathrm{STMHT}}}={\left\{ \begin{array}{ll} -1 & \text {if}\quad { HS }_{\mathrm{STMHT}}<0,\\ 0 & \text {if} \quad { HS }_{\mathrm{STMHT}}=0,\\ +1 & \text {if} \quad { HS }_{\mathrm{STMHT}}>0. \end{array}\right. } \end{aligned}$$
- ${\textcircled {{2}}}$ Then, the variation in $S_{{ HS }_{\mathrm{STMHT}}}$ (${ DS }_{{ HS }_{\mathrm{STMHT}}}$) is determined from Eq. (6)
  6 $$\begin{aligned} { DS }_{{ HS }_{\mathrm{STMHT}}}[i]=S_{{ HS }_{\mathrm{STMHT}}}[i+1]-S_{{ HS }_{\mathrm{STMHT}}}[i], i=1,2,\ldots ,n. \end{aligned}$$
- ${\textcircled {{3}}}$ Finally, **N2P** and **P2N** are determined by
  7 $$\begin{aligned} {\left\{ \begin{array}{ll} {\mathrm{N2P}}=i/{F_{\mathrm{S}}} & \text {if}\quad { DS }_{{ HS }_{\mathrm{STMHT}}}[i]=+2\\ {\mathrm{P2N}}=i/{F_{\mathrm{S}}} & \text {if}\quad { DS }_{{ HS }_{\mathrm{STMHT}}}[i]=-2 \end{array}\right. } \end{aligned}$$

**(2)**: **Automatic extraction of** ${ CS }_{1}$ **and** ${ CS }_{2}$

- ${\textcircled {{1}}}$ Calculate the difference between two adjacent **N2P**s, denoted as $D_{\mathrm{N2P}}$, with Eq. (8)
  8 $$\begin{aligned} D_{_\mathrm{N2P}}[i]={\mathrm{N2P}}[i+1]-{\mathrm{N2P}}[i], i=1,2,\ldots ,n. \end{aligned}$$
- ${\textcircled {{2}}}$ Determine the points ${CS}_{\mathrm{12}}$ and ${CS}_{\mathrm{21}}$ that are used for segmentation from ${ CS }_{1}$ to ${ CS }_{2}$ and from ${ CS }_{2}$ to ${ CS }_{1}$, respectively, by using Eq. (9).
  9 $$\begin{aligned} {\left\{ \begin{array}{ll} { CS }_{21}[i]={\mathrm{P2N}}[i], {\mathrm{CS}}_{12}[i]={\mathrm{P2N}}[i+1], & \text {if}\quad D_{_\mathrm{N2P}}[i]< D_{_\mathrm{N2P}}[i+1]\\ { CS }_{21}[i]={\mathrm{P2N}}[i+1], {\mathrm{CS}}_{12}[i]={\mathrm{P2N}}[i+2], & \text {Otherwise} \end{array}\right. }. \end{aligned}$$
- ${\textcircled {{3}}}$ Extract $CS _{1}$ (denoted as ${{ CS }_{1}}_{i}$) and $CS _{2}$ (denoted as ${{ CS }_{2}}_{i}$) for the *i*th period of an **HS** as follows
  10 $$\begin{aligned} {\left\{ \begin{array}{ll} {{ CS }_{1}}_{i}={ HS }_{\mathrm{T}}[{ CS }_{21_{i}}:{ CS }_{12_{i}}]\\ {{ CS }_{2}}_{i}={ HS }_{\mathrm{T}}[{ CS }_{12_{i}}:{ CS }_{21_{i+1}}] \end{array}\right. } \end{aligned}$$

The automatic extraction procedures for ${ CS }_{1}$ and ${ CS }_{2}$ are illustrated in Fig. 3. Figure 3(A, B) show a typical **AR** sound, and the typical **NM** sound is shown in Fig. 3(C, D).

**Figure 4:**
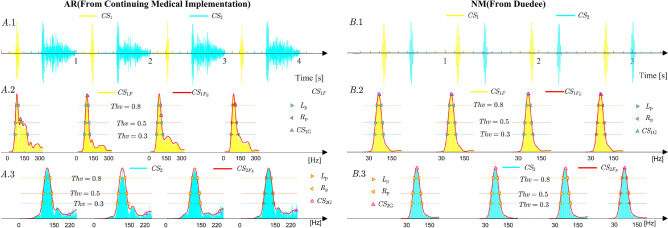
Example of feature definition and automatic extraction.

### Stage 2: Automatic feature generation

#### Feature definition

To extract the efficient frequency widths, as shown in Fig. 4, the smooth envelopes for ${{CS}}_{1}$ and ${{CS}}_{2}$ in the frequency domain are firstly generated, and then the frequency widths corresponding to different Thv values are extracted.

(**1**): **Secondary envelopes** ${{ CS }_{1}}_{ F _{\mathrm{E}}}$ **and** ${{ CS }_{2}}_{ F _{\mathrm{E}}}$ **generation:** Given an *M*-point **HS**, the secondary envelope in the frequency-domain, denoted as ${ HS }_{\mathrm{F}}$, can be calculated from Eq. (11):
  11 $$\begin{aligned} { HS }_{ F _{\mathrm{E}}}[k]=\frac{\sum _{l_{1}}(L_{1}+L_{2}+1-|l_{1}|){ HS }_{{\mathrm{F}}}[k+l_{1}]-\sum _{l_{2}}{ HS }_{{\mathrm{F}}}[k+l_{2}]}{(2L_{1}+1)(2L_{2}+1)}, \end{aligned}$$
  where $l_{1}$, $l_{2}$ and ${ HS }_{\mathrm{F}}[k]$ are defined by Eq. (12):
  12 $$\begin{aligned} {\left\{ \begin{array}{ll} l_{1}=-(L_{1}+L_{2}),-(L_{1}+L_{2})+1,\ldots , (L_{1}+L_{2})\\ l_{2}=-(L_{1}-L_{2}-1),-(L_{1}-L_{2}-1)+1,\ldots , (L_{1}-L_{2}-1)\\ { HS }_{\mathrm{F}}[k]=\left| \sum _{m=0}^{\mathrm{M-1}}{ HS _{T}}[m+1]e^{(-j\frac{2\pi kl}{{\mathrm{M}}})}\right| , \quad k=0,1,2,\ldots ,M-1 \end{array}\right. }, \end{aligned}$$
  $|\cdot |$ is the absolute value sign, $2L_{1}+1$ is the first window width, and $2L_{2}+1$ is the second window width. According to studies^9,28^, $L_{1}$ and $L_{2}$ are set to 9 and 17, respectively. Moreover, $HS _{{ F }_{\mathrm{E}}}$ is also normalized by setting the maximum amplitude of $HS _{{ F }_{\mathrm{E}}}$ to 1. The secondary envelopes for ${ CS }_{1}$ and ${ CS }_{2}$, denoted as ${{ CS }_{1}}_{ F _{\mathrm{E}}}$ and ${{ CS }_{2}}_{ F _{\mathrm{E}}}$ respectively, are illustrated by using the examples described in Fig. 3 which are first automatically generated based on Eq. (11), are shown in Fig. 4, where the plots in Fig. 4A.2 describe the results of ${{ CS }_{1}}_{ F _{\mathrm{E}}}$ corresponding to ${ CS }_{1}$ in Fig. 4A.1, the plots in Fig. 4B.2 describe the results of ${{ CS }_{1}}_{ F _{\mathrm{E}}}$ which corresponds to ${ CS }_{1}$ in Fig. 4B.1, the plots in Fig. 4A.3 describe the results of ${{ CS }_{2}}_{ F _{\mathrm{E}}}$ corresponding to ${ CS }_{2}$ in Fig. 4A.1, and the plots in Fig. 4B.3 describe the results of ${{ CS }_{1}}_{ F _{\mathrm{E}}}$ which corresponds to ${ CS }_{2}$ in Fig. 4B.1.
(**2**): **Definition and automatic extraction of frequency features:** The frequency features are illustrated in Fig. 4(B, C), and their gravities are calculated by
  13 $$\begin{aligned} {\left\{ \begin{array}{ll} {{ CS }_{1}}_{{\mathrm{G}}}=\frac{\sum _{k=0}^{M-1}{k\times { CS _{1}}_{ F _{\mathrm{E}}}[k]}}{\sum _{k=0}^{M-1}{{ CS _{1}}_{ F _{\mathrm{E}}}[k]}}\\ {{ CS }_{2}}_{{\mathrm{G}}}=\frac{\sum _{k=0}^{M-1}{k\times { CS _{2}}_{ F _{\mathrm{E}}}[k]}}{\sum _{k=0}^{M-1}{{ CS _{2}}_{ F _{\mathrm{E}}}[k]}}\\ \end{array}\right. }, \end{aligned}$$

The frequency widths over a given threshold value are defined and calculated by

14 $$\begin{aligned} {\left\{ \begin{array}{ll} { CS _{1}}_{\mathrm{FWi}}={R_{p}}_{i}-{L_{p}}_{i}\\ { CS _{2}}_{\mathrm{FWi}}={R_{p}}_{i}-{L_{p}}_{i} \end{array}\right. }, i=1,2,3. \end{aligned}$$

where ${L_{p}}_{i}$ and ${R_{p}}_{i}$ are the $i^{th}$ left and right intersections, respectively, of ${{ CS }_{1}}_{{ F }_{\mathrm{E}}}$ and ${{ CS }_{2}}_{{ F }_{\mathrm{E}}}$ over the $Thv$ lines (${ Thv }$=0.3, 0.5 and 0.8). Moreover, the frequency features are expressed based on Eq. (15) and described in Table 1.

15 $$\begin{aligned} \mathrm{FF}=[{ CS _{1}}_{\mathrm{FW1}},{ CS _{1}}_{\mathrm{FW2}},{ CS _{1}}_{\mathrm{FW3}},{ CS _{1}}_{\mathrm{G}}, { CS _{2}}_{\mathrm{FW1}},{ CS _{2}}_{\mathrm{FW2}},{ CS _{2}}_{\mathrm{FW3}},{ CS _{2}}_{\mathrm{G}}] \end{aligned}$$

**Table 1:** Description of the frequency domain feature matrix $\mathrm{FF}$.

Feature index	Feature’s symbol	Feature description	Unit
1	${ CS _{1}}_{\mathrm{FW1}}$	The frequency width of ${ CS }_{1}$ corresponding to ${Thv}=0.3$	Hz
2	${ CS _{1}}_{\mathrm{FW2}}$	The frequency width of ${ CS }_{1}$ corresponding to ${Thv}=0.5$	Hz
3	${ CS _{1}}_{\mathrm{FW3}}$	The frequency width of ${ CS }_{1}$ corresponding to ${Thv}=0.8$	Hz
4	${ CS _{1}}_{\mathrm{G}}$	The Center of gravity of ${ CS }_{1}$ in frequency-domain	Hz
5	${ CS _{2}}_{\mathrm{FW1}}$	The frequency width of ${ CS }_{2}$ corresponding to ${Thv}=0.3$	Hz
6	${ CS _{2}}_{\mathrm{FW2}}$	The frequency width of ${ CS }_{2}$ corresponding to ${Thv}=0.5$	Hz
7	${ CS _{2}}_{\mathrm{FW3}}$	The frequency width of ${ CS }_{2}$ corresponding to ${Thv}=0.8$	Hz
8	${ CS _{2}}_{\mathrm{G}}$	The Center of gravity of ${ CS }_{2}$ in frequency-domain	Hz

#### Experimental results for several typical types of heart disease

**Figure 5:**
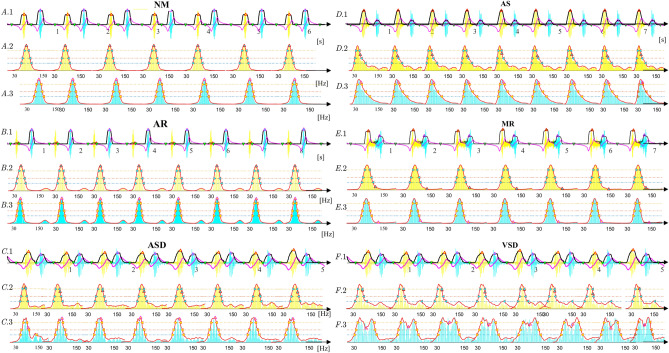
Examples of a typical normal sound and six types of typical heart disease sounds.

**Figure 6:**
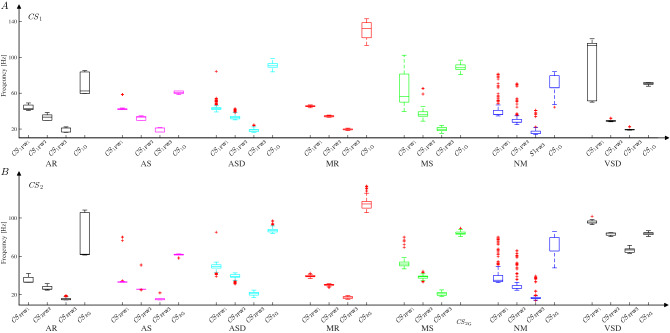
Box plot representation of FF for each type of heart disease. ***A*** shows the box plots for features from ${ CS }_{1}$. In addition, ***B*** represents the features from ${ CS }_{2}$.

The features $\mathrm{FF}$ of six typical and normal sounds are illustrated in Fig. 5. From Fig. 5, ${ CS }_{1}$ and ${ CS }_{2}$ are first automatically located and extracted, then, the envelopes for every ${ CS }_{1}$ and ${ CS }_{2}$ are extracted by Eq. (11). Finally, the features defined by Eq. (15) for ${ CS }_{1}$ and ${ CS }_{2}$ in the frequency domain are automatically extracted with Eqs. (13-14). The experimental sounds are 665-period **AR** sounds (3M database^29^, medical sound library^30^, heart auscultation sounds^31^, auscultation sound^32^, continuing medical implementation^26^, sounds Database of the University of Dundee^27^, and patients only with **AR** disease from the Nanyang First People’s Hospital), 381-period **AS** sounds (continuing medical implementation^26^, sounds database of the University of Dundee^27^, 3M database^29^, medical sound library^30^, auscultation sound^32^, and patients only with **AS** disease from the Nanyang first People’s Hospital, and heart auscultation sounds^31^), 315-period **ASD** sounds (Medical sound library^30^, heart auscultation sounds^31^, 3M database^29^, patients only with **ASD** disease from the Nanyang First People’s Hospital, and medical sound library^30^), 769-period **MR** sounds (3M database^29^, sounds database of the University of Dundee^27^, heart auscultation sounds^31^, medical sound library^30^, and auscultation sound^32^), 439-period **MS** sounds(3M database^29^, auscultation sound^32^, medical sound library^30^, and continuing medical implementation^26^), and 1056-period **NM** sounds(3M database^29^, Michigan database^33^, medical sound library^30^, ThinkLabs database^34^, and healthy undergraduates from Nanyang Institute of Technology, China)(whom I thank for the data used in this study)). Moreover, the boxplots for the features are plotted in Fig. 6, where Fig. 6A shows the features extracted from ${ CS }_{1}$ and Fig. 6B shows the features from ${ CS }_{2}$ for each type of heart disease. The scatter plots of features in Fig. 6 illustrate the discrimination ability of the model in distinguishing among different heart diseases and highlighting the following findings: ${\textcircled {{1}}}$ The **MS** and **VSD** sounds are easy to distinguish from the other sounds by using ${{ CS }_{1}}_{\mathrm{FW1}}$ (Fig. 6A), and by using the ${{ CS }_{2}}_{\mathrm{FW1}}$ (Fig. 6B), the **VSD** sound is easy to distinguish from the other sounds; ${\textcircled {2}}$ The **MS** sound is easy to distinguish from the other sounds based on ${{ CS }_{1}}_{\mathrm{FW2}}$ (Fig. 6A), and by using the ${{ CS }_{2}}_{\mathrm{FW2}}$ (Fig. 6B), the **AR** and **VSD** sounds are distinguished from other sounds; ${\textcircled {3}}$ The **NM** sound is easy to distinguish from other sounds using ${{ CS }_{1}}_{\mathrm{FW3}}$ (Fig. 6A). ${\textcircled {4}}$ The **AR** and **VSD** sounds are easy to distinguish from the other sounds using ${{ CS }_{2}}_{\mathrm{FW3}}$, as shown in Fig. 6B; ${\textcircled {5}}$ Fig. 6A indicates that ${{ CS }_{1}}_{\mathrm{G}}$ can be used to easily distinguish **MR** from other sounds and the **AS** and **ASD** sounds from other sounds; ${\textcircled {6}}$ Fig. 6B shows that the distribution of ${{ CS }_{2}}_{\mathrm{G}}$ from **AS** sounds is different from that for other sounds, except **NM** sounds. The analysis results discussed above indicate that different combinations of several features defined by Eq. (15) can be used to distinguish among various types of heart disease. Therefore, to simplify features and develop a diagnostic method that is simple and effective, dimension reduction is used to determine new features; this process is described in detail as follows. 
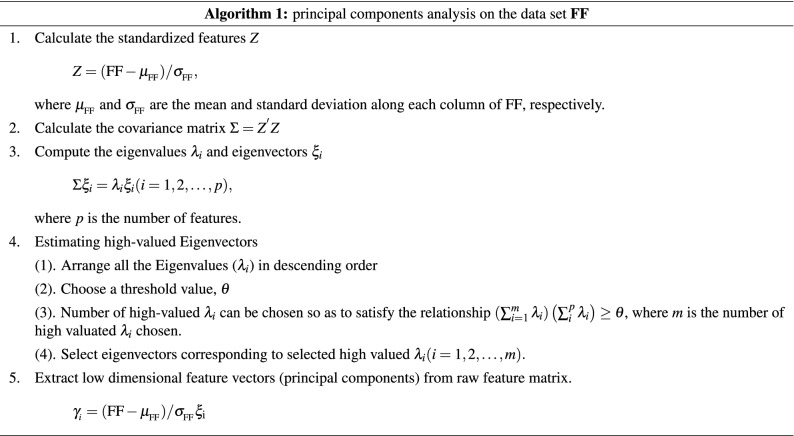

#### Diagnostic feature determination

**Figure 7:**
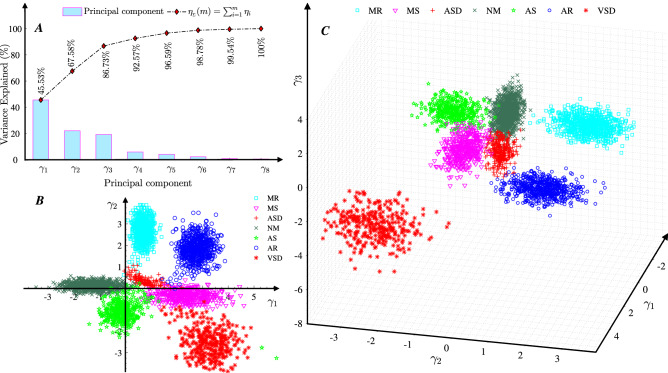
PCA results. **A** shows the Pareto chart of the variance by contribution of each principal component, **B** plots the scatter diagram of the first two components $\gamma _{1}$ and $\gamma _{2}$, and **C** shows the first three components $\gamma _{1}$, $\gamma _{2}$ and $\gamma _{3}$.

To simplify the computation when using features to diagnose heart diseases, **PCA**, a linear dimensionality reduction technique for finding principal components and replacing high-dimension data in many studies, such as studies on heart arrhythmias classification^35^, heart disease classification^2,36^, emotion recognition^37^, respiratory rate extraction^38^ and electrocardiogram heart disease diagnosis^39^, is employed to generate a few efficient principal components to characterize **HS** features and diagnose heart diseases. The algorithm corresponding to the generation of new features via **PCA** for a given data set **FF** is described as **Algorithm 1**. The eigenvector $\xi _{i}$ in **Algorithm 1** , which corresponds to the eigenvalue $\lambda _{i}$ and is calculated for the matrix *Z* in step 2, as shown in Table 3, is the actual weighted coefficient for the *i*th principal component $\gamma _{i}$. Table 3 shows that the largest absolute coefficients in the first principal component $\gamma _{1}$ are ${{ CS }_{1}}_{\mathrm{FW1}}$, ${{ CS }_{2}}_{\mathrm{FW2}}$ and ${{ CS }_{1}}_{\mathrm{FW2}}$; the second principal component $\gamma _{2}$ is mainly weighted based on ${{ CS }_{1}}_{\mathrm{G}}$, ${{ CS }_{2}}_{\mathrm{FW2}}$, ${{ CS }_{1}}_{\mathrm{FW1}}$ and ${{ CS }_{2}}_{\mathrm{FW3}}$; and the third component $\gamma _{3}$ is mainly weighted based on ${{ CS }_{1}}_{\mathrm{FW2}}$, ${{ CS }_{1}}_{\mathrm{FW3}}$ and ${{ CS }_{2}}_{\mathrm{FW3}}$ (Table 3). To determine the smallest number of principal components *m* should be considered, the Pareto chart is used; this chart provides a tool for visualizing the Pareto principle, which states that observing a small set of variables that influence a common outcome is more common than detecting many variables that influence the same outcome. This approach has been used to determine the percent variability explained by each principal component (Fig. 7A). Therefore, according to the smallest *m* value such that $\eta _{_\Sigma }(m)> 80\%$^40^, combined with the scatter plot for the first *m* principal components, the smallest *m* is determined. The Pareto chart of the **PCA** results in Fig. 7A shows the explained variance and accumulated variance for each principal component $\gamma _{i}$, where $i=1, 2, \ldots , 8$. According to Fig. 7A, $67.58\%$ of the total variance is captured by the first two components, $\gamma _{1}$ and $\gamma _{2}$, and $86.73\%$ of the total variance is captured by the first three components $\gamma _{1}$, $\gamma _{2}$ and $\gamma _{3}$. Therefore, the following conclusions can be obtained.

- $\gamma _{1}$ and $\gamma _{2}$ lead to a dimensionality reduction of $75\%$ (from 8 to 2 variables) and only $32.42\%$ information loss. The scatter diagram of $\gamma _{1}$ and $\gamma _{2}$ given in Fig. 7B indicates that although the distribution region corresponding to each type of heart disease is obviously different and the overlaps between **MR** and other diseases, **AR** and other diseases, and **VSD** and other diseases are small, the overlaps among **MS**, **ASD**, **NM**, and **AS** are relatively large; therefore, it is difficult to accurately distinguish among these four types of heart diseases.
- However, the scatter diagram of $\gamma _{1}$, $\gamma _{2}$ and $\gamma _{3}$, plotted in Fig. 7C, shows that there are different distribution regions for these types of heart diseases. In addition, $\eta _{\Sigma }(3)=86.73\%$, as shown in Fig. 7A, based on feature number determination^40^. Thus, $\gamma _{1}$, $\gamma _{2}$ and $\gamma _{3}$ lead to a dimensionality reduction of $62.5\%$ (from 8 to 3 variables) with only $13.27\%$ information loss. The scatter diagram of $\gamma _{1}$, $\gamma _{2}$ and $\gamma _{3}$ in Fig. 7C is used to verify the different distribution regions corresponding to these types of heart diseases.

Therefore, *m* is set to 3, and the new 3-dimensional feature matrices consisting of $\gamma _{1}$, $\gamma _{2}$ and $\gamma _{3}$ (see Fig. 7C) are used to diagnose heart diseases.

**Table 2:** Mean ($\mu _{_\mathrm{FF}}$) and standard deviation ($\sigma _{_\mathrm{FF}}$) of the features.

Statistics	Frequency features ($\mu _{_\mathrm{FF}}+\sigma _{_\mathrm{FF}}$)							
	Features from ${{ CS }_{1}}$				Features from ${ CS }_{2}$			
	${{ CS }_{1}}_{\mathrm{FW1}}$	${{ CS }_{1}}_{\mathrm{FW2}}$	${{ CS }_{1}}_{\mathrm{FW3}}$	${{ CS }_{1}}_{\mathrm{G}}$	${{ CS }_{2}}_{\mathrm{FW1}}$	${{ CS }_{2}}_{\mathrm{FW2}}$	${{ CS }_{2}}_{\mathrm{FW3}}$	${{ CS }_{2}}_{\mathrm{G}}$
$\mu _{_\mathrm{FF}} \pm \sigma _{_\mathrm{FF}}$	45.3± 11.8	33.1± 5.8	18.8± 3.6	80.6± 21.7	44.1± 23.1	32.2± 9.1	$18.4 \pm 6.5$	79.8±18.9

**Table 3:** Eigenvector $\xi _{i}$ and eigenvalue $\lambda _{i} (i = 1,\ldots ,8)$ for $\Sigma$ in descending order of eigenvalues.

Features	Eigenvector (eigenvalue) in descending order of eigenvalues							
	$\xi _{1}(\lambda _{1}=3.6423)$	$\xi _{2}(\lambda _{2}=1.7643)$	$\xi _{3}(\lambda _{3}=1.5316)$	$\xi _{4}(\lambda _{4}=0.4671)$	$\xi _{5}(\lambda _{5}=0.3226)$	$\xi _{6}(\lambda _{6}=0.1748)$	$\xi _{7}(\lambda _{7}=0.0606)$	$\xi _{8}(\lambda _{8}=0.0368)$
${{ S }1}_{\mathrm{FW1}}$	0.4309	-0.2169	0.0818	-0.2179	0.8062	-0.2394	-0.0499	0.0616
${{ S }1}_{\mathrm{FW2}}$	0.3716	-0.0757	0.5303	0.0431	-0.0306	0.6778	0.2873	-0.1735
${{ S }1}_{\mathrm{FW3}}$	0.3411	-0.0983	0.5431	0.0444	-0.4364	-0.6126	-0.1001	-0.0375
${{ S }1}_{\mathrm{G}}$	0.2385	0.6501	-0.0157	-0.2122	0.0212	0.0986	-0.5500	-0.4031
${{ S }2}_{\mathrm{FW1}}$	0.3313	0.0487	-0.2471	0.8958	0.0977	-0.0299	-0.0699	-0.0944
${{ S }2}_{\mathrm{FW2}}$	0.4475	-0.2390	-0.2924	-0.1628	-0.3044	0.2633	-0.3928	0.5607
${{ S }2}_{\mathrm{FW3}}$	0.3517	-0.2191	-0.5142	-0.2650	-0.2357	-0.1047	0.3750	-0.5353
${{ S }2}_{\mathrm{G}}$	0.2632	0.1026	-0.0768	-0.0662	-0.0207	-0.1308	0.5506	0.4386

**Figure 8:**
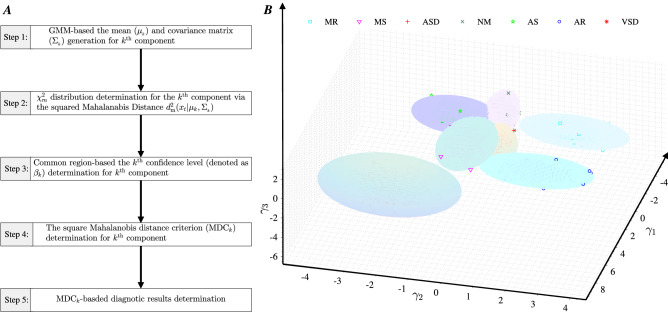
Flow chart of the diagnostic determination and 3-dimensional surface classifier results.

### Stage 3: classification based on the squared Mahalanobis distance criterion

#### Classifier determination

The squared Mahalanobis distance classification criterion-based diagnostic methodology, consisting of the five sequential steps as shown in the flow chart (Fig. 8A), is proposed to diagnose **HS**s and is described in the following 5 steps.

#### Step 1: GMM-based $\mu _{_k}$ and $\Sigma _{_k}$ generation

In the design step of **GMM**, the estimated target function, ${f_{{ et}}(\mathbf{x}) }$, is a mixture of *d*-dimensional normal Gaussian distributions ${p({{\mathbf {x}}}|\mu _{_{k}},\Sigma _{_{k}})}$ that reflect the training pattern of each component; it is assumed that components can be modeled by mixtures of normal Gaussian distributions by

19 $$\begin{aligned} f_{{ et}}({\mathbf{x}})=\sum _{k=1}^{K}{\pi _{_{k}}p({\mathbf{x}}|\mu _{_{k}},\Sigma _{_{k}}),} \end{aligned}$$

where

20 $$\begin{aligned} p({\mathbf{x}})=\frac{1}{\sqrt{(2\pi )^{d}\mid \Sigma _{_{k}}\mid }}\exp ^{\left( -\frac{1}{2}({\mathbf{x}}-\mu _{_{k}})^{\mathrm{T}}\Sigma _{_{k}}^{-1}({\mathbf{x}}-\mu _{_{k}})\right) } \end{aligned}$$

expresses the posterior probabilities corresponding to each component; *K* is the number of components; $\pi _{_{k}}$ corresponds to the mixed weights, such that $\sum _{k=1}^{K}{\pi _{_{k}}}=1$; and $\mu _{_{k}}$ and $\Sigma _{_{k}}$ are the mean value and covariance matrix of the $k^{\mathrm{th}}$ component, respectively. Because the goal is to maximize the function $f_{{ et}}({\mathbf{x }})$, the parameters ($\pi _{_k}$, $\mu _{_k}$, and $\Sigma _{_k}$) are determined based on the **EM** algorithm^41^ for a set of sample records. Based on the types of heart disease described in Sect. 2.2 and the scatter diagram plotted in Fig. 7C, the number of Gaussian mixture components is set to $K=7$, and the *fitgmdist* function in MATLAB 2018b is used to return a **GMM** with $K=7$ components fitted to the features $[\gamma _{_{1}}, \gamma _{_{2}}, \gamma _{_{3}}]$ established in Sect. 2.2 using the **EM** algorithm by assigning a posterior probability to each component density with respect to each observation. Furthermore, the regularization value is set as 0.01 to avoid ill-conditioned covariance estimates, and the number of optimization iterations is set to 1000 based on experience. The Gaussian mixture parameter estimates for $\pi _{_{k}}, \mu _{_{k}}$ and $\Sigma _{_{k}}$ are obtained and shown in Table 4. To characterize the 3-dimensional interspace corresponding to each 3-dimensional Gaussian component for diagnosing heart diseases, the 3-dimensional interspaces can be used as 3-dimensional classifiers to diagnose heart diseases with high classification accuracy; the overlapping interspace between two random components is made as small as possible, and the independent 3-dimensional interspace corresponding to each component is considered.

**Table 4:** The Gaussian mixture parameter estimates are achieved for the new features $[\gamma _{_{1}}, \gamma _{_{2}}, \gamma _{_{3}}]$ by setting the number of Gaussian mixture components as 7.

Components	Component number	Gaussian mixture parameter estimates						
		$\pi _{_k}$		$\mu _{k}$		$\Sigma _{k}$		
**MR** Classifier	$k=1$	0.1947	0.7056	2.7126	1.4950	0.0425	-0.0007	0.0013
						-0.0007	0.2343	-0.0126
						0.0013	-0.0126	0.2122
**MS** Classifier	$k=2$	0.0827	3.2981	-2.6064	-3.7382	0.3310	-0.0094	-0.0122
						-0.0094	0.3906	-0.0210
						-0.0122	-0.0210	0.5386
**ASD** Classifier	$k=3$	0.1130	2.3453	-0.3484	0.5773	0.5373	-0.0172	-0.0039
						-0.0172	0.0608	-0.0053
						-0.0039	-0.0053	0.1883
**NM** Classifier	$k=4$	0.1683	2.7874	1.8620	-0.9829	0.1403	0.0107	0.0063
						0.0107	0.2549	0.0016
						0.0063	0.0016	0.1301
**AS** Classifier	$k=5$	0.0783	0.7511	0.3199	-0.5341	0.0972	0.0077	-0.0161
						0.0077	0.0344	-0.0050
						-0.0161	-0.0050	0.2634
**AR** Classifier	$k=6$	0.2676	-1.2294	0.1198	0.3222	0.3301	-0.0011	0.0025
						-0.0011	0.0230	0.0005
						0.0025	0.0005	0.3255
**VSD** Classifier	$k=7$	0.0954	-0.1631	-1.1167	0.9454	0.1338	0.0048	-0.0155
						0.0048	0.1449	-0.0095
						-0.0155	-0.0095	0.1573

#### Step 2: $\chi _{_3}^{2}$ determination for the $k^{\mathrm{th}}$ component in 3-dimensional interspace

Since the squared Mahalanobis distances for each Gaussian component follow the Chi-square distribution ($\chi _{3}^{2}$) in 3-dimensional interspace, to determine the decision region for classifying the test data **x** via the components estimated in the above step, the squared Mahalanobis distance in 3-dimensional interspace for the $k^{\mathrm{th}}$ component with mean $\mu _{_{k}}$ and full covariance matrix $\Sigma _{_{k}}$, $d_{_3}^2({\mathbf{x}}| \mu _{_{k}}, \Sigma _{_{k}})$, is computed as follows:

21 $$\begin{aligned} d_{_3}^{2}({\mathbf{x}}| \mu _{_{k}}, \Sigma _{_{k}})=({\mathbf{x}}-\mu _{_{k}})^{\mathrm{T}}\Sigma _{_{k}}^{-1}({\mathbf{x}}-\mu _{_{k}}) . \end{aligned}$$

Therefore, $\chi _{3}^{2}$, which is constructed based on component *k* and denoted as $\chi _{3}^{2}(\mu _{_{k}}, \Sigma _{_{k}})$, is determined by

22 $$\begin{aligned} d_{3}^{2}({\mathbf{x}}| \mu _{_{k}}, \Sigma _{_{k}})\sim \chi _{3}^{2}(\mu _{_k},\Sigma _{_{k}}). \end{aligned}$$

Therefore, the squared Mahalanobis distance $d_{3}^{2}({\mathbf{x}}| \mu _{_{k}}, \Sigma _{_{k}})$ specified based on the desired confidence level, denoted as $\beta _{_k}$, can be used as the *k*th classifier criterion for determining whether feature **x** belongs to the *k*th class.

**Figure 9:**
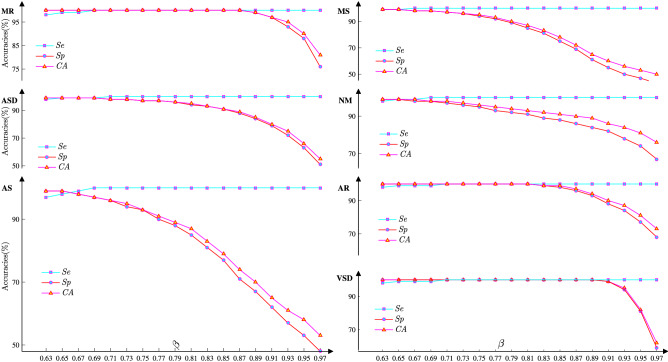
The achieved accuracies corresponding to classifying the heart sounds described in Sect. 2.2 by setting $\beta$ form 0.63 to 0.97 with a step of 0.02.

#### Step 3: The $k^{\mathrm{th}}$ confidence level $(\beta _{_k})$ determination

Actually, the *k*th confidence region, as specified by the *k*th desired confidence level $\beta _{_{k}}$, is surrounded by the *k*th ellipsoid, and this relation is expressed as

23 $$\begin{aligned} d_{3}^{2}({\mathbf{x}}| \mu _{_{k}}, \Sigma _{_{k}})\le {\mathrm{MDC}}_{_k}={{{\chi ^{2}}}_{3,\beta _{_{k}}}}, \end{aligned}$$

where ${{\chi }^{2}_{3, \beta _{_{k}}}}$ is the inverse of $\chi _{3}^{2}$ for a given confidence level $\beta _{_k}$, and ${\mathrm{MDC}}_{{k}}$ represents the classification criterion for component *k* and satisfies the following equation

24 $$\begin{aligned} \chi ^{2}\big (d_{\mathrm{3}}^{2}({\mathbf{x}}| \mu _{_{k}}, \Sigma _{_{k}})={\mathrm{MDC}}_{_k}\big |\mu _{_{k}},\Sigma _{_k}\big )=\beta _{_{k}} \end{aligned}$$

For the $\chi _{3}^{2}$ distribution, although the confidence regions corresponding to the confidence levels of $68.3\%$, $95\%$, and $97.5\%$ are widely used classification criteria in many studies^2,42–45^, the optional $\beta _{_k}$ is identified by setting $\beta _{k}\in [63:2:97]\%$ combined with the following rules: 1) each ellipsoid should be as large as possible; 2) each common region should be as small as possible; and 3) the classification accuracy defined in Eq. (26) should be as high as possible. The classification accuracies for classifying sound data summarized in Sect. 2.2 are plotted in Figs. 9, and 9 shows the following results: ${\textcircled {1}}$ For **VSD** sounds, high accuracy can be achieved by setting the desired confidence level $\beta$ to each value within the interval of ($0.71<\beta <0.89$), as shown in Fig. 9(**VSD**); ${\textcircled {2}}$ For **AR** and **MR** sounds, by setting the desired confidence level $\beta$ based on $\beta \in [0.69,0.81]$, high classification accuracy could be achieved (Fig. 9(**MR** and **AR**) ); ${\textcircled {3}}$ For **MS**, **AS** and **NM** sounds, to achieve the accurate classification of **HS**s, the interval of the desired confidence level $\beta$ should be set as [0.63, 0.65] (Fig. 9); ${\textcircled {4}}$ For **ASD** sounds, Fig. 9(**ASD**) shows that the highest classification accuracy is achieved by setting the desired confidence level to $\beta \in [0.63, 0.69]$. Furthermore, the desired confidence level $\beta$ can be adjusted to improve the classification accuracy and fit new datasets without reperforming the computations for the objective function, especially for **VSD** sounds and **MR** sounds (Fig. 9(**VSD** and **MR**)). In this study, according to the rules described above combined with the accuracy analysis results plotted in Fig. 9, the $\beta _{k}(k=1, \ldots , 7)$ values are set as 0.87, 0.65, 0.67, 0.65, 0.67, 0.79 and 0.87, respectively.

#### Step 4: ${\mathrm{MDC}}_{_k}$ determination corresponding to $\beta _{_k}$

Based on the $k^{\mathrm{th}}$ confidence level achieved for $\beta _{_k}$ in the above step, by using the function ’*chi*2*inv*’ in MATLAB 2018b, the inverse of $\chi ^{2}_{_{3,\beta {_{_k}}}}$, denoted as ${\mathrm{MDC}}_{_k}= {\chi ^{2}}_{{3,\beta _{_{k}}}}$, is determined. The analysis results for the $k^{\mathrm{th}}$ confidence region in the 3-dimensional interspace, which is surrounded by the $k^{\mathrm{th}}$ ellipsoid corresponding to the $k^{\mathrm{th}}$ desired confidence level $\beta _{_{k}}$ , are determined and shown in Fig. 8B. Furthermore, Fig. 8B shows that the common regions between two random ellipsoids are almost zero; thus, a faulty decision process is avoided because the input will not fall into two or more categories.

#### Step 5: $\mathrm{MDC}_{_k}$-based diagnostic result determination

Based on the ellipsoid surfaces region shown in Fig. 8B, the diagnosis method is described as follows.

- ${\textcircled {{1}}}$ The 3-dimensional diagnostic features [$\gamma _{1}$, $\gamma _{2}$, $\gamma _{3}$] are first transformed from the features FF (denoted as ${\mathrm{FF}_{s}}$) of the testing sample and calculated with the following equation
  25 $$\begin{aligned} \gamma _{i}=\frac{{\mathrm{FF}}_{s}-\mu _{_\mathrm{FF}}}{\sigma _{_\mathrm{FF}}}\times \xi _{i}, i=1,2,3 \end{aligned}$$
  where $\mu _{_\mathrm{FF}}$ and $\sigma _{_\mathrm{FF}}$ are shown in Table 2.
- ${\textcircled {{2}}}$ Then, according to the confidence region shown in Fig. 8B, the $\mathrm{MDC}_{_k}$-based diagnostic result for a test features $\mathbf{x }=[\gamma _{_1}, \gamma _{_2}, \gamma _{_{3}}]$ is determined.
- ${\textcircled {{3}}}$ $\mathrm{MDC}_{_k}$-based diagnostic results for a test feature ${\mathbf{x }}=[\gamma _{_1}, \gamma _{_2}, \gamma _{_{3}}]$ are determined by
  26 $$\begin{aligned} {\left\{ \begin{array}{ll} {\text {Class }}k, d_{3}^{2}(\mathbf{x}| \mu _{_{k}}, \Sigma _{_{k}})\le {\mathrm{MDC}}_{_{k}} \\ \text {Unknown class}, \quad \quad \text {otherwise} \end{array}\right. }, \end{aligned}$$
  where class *k* corresponding to the type of heart disease is detailed in Table 4, and ${\mathrm{MDC}}_{_{k}}$ (1, 2, $\ldots$, 7) is 5.6489, 3.2831, 3.4297, 3.2831, 3.4297, 4.5258 and 5.6489.

#### Performance evaluation criteria

To evaluate the performance of these ellipsoids in 3-dimensional space, the classification accuracy ($CA$), sensitivity ($Se$) and specificity ($Sp$) values are calculated by

27 $$\begin{aligned} {\left\{ \begin{array}{ll} { CA }(\%)&=\frac{{\mathrm{TP}}+{\mathrm{TN}}}{\mathrm{TP+FP+FN+TN}}\times 100 \\ { Se }(\%)&=\frac{\mathrm{TP}}{\mathrm{TP+FN}}\times 100\\ { Sp }(\%)&=\frac{\mathrm{TN}}{\mathrm{FP+TN}}\times 100 \end{array}\right. }, \end{aligned}$$

where $\mathrm{TP}$, $\mathrm{FP}$, $\mathrm{TN}$ and $\mathrm{FN}$ are the numbers of true positives, false positives, true negatives and false negatives, respectively.

**Table 5:** Experimental sounds used to evaluate the performance.

Data source	Period numbers of every type of heart disease/Patients						
	**MR**	**MS**	**ASD**	**NM**	**AS**	**AR**	**VSD**
Sounds in Sect. 2.2	769/10	439/5	315/7	1056/45	381/10	665/15	327/10
New sounds	156/3	132/2	82/2	183/8	126/3	153/4	70/3
Total sounds	925/13	571/7	397/9	1239/53	507/13	818/19	397/13

## Performance evaluation

To evaluate the performance of the proposed methodology, the comparison between the proposed methodology and the state-of-the-art methods on the clinical sounds and online sounds data was conducted as follows.

- $\bullet$ Total sounds: The total sounds, consisting of sounds described in Sect. 2.2 and new sounds, were summarized in Table 5 to evaluate the performance of this proposed methodology.
- $\bullet$ State-of-the-art methods: To highlight the efficiency of the proposed methodology for diagnosing the seven typical heart diseases, the state-of-the-art methods, published in recent five years and described in Table 6, were comparatively analyzed.
- $\bullet$ Comparsion results: The comparison results were summarized in Table 7, where the parameters corresponding to the state-of-the-art methods were described in Table 8. The results in Table 7 support the following conclusions.
- $\bullet$ Although using the method $\#1$ to diagnose **AS** yielded a higher ${ Sp }$ than that of the proposed method, the ${ CA }$ was lower than that of the proposed method, partially due to the high ${ Se }$ achieved by the proposed method.
- $\bullet$ Although using the method $\#3$ to diagnose **MS** yielded a higher ${ Sp }$ than that of the proposed method, the ${ CA }$ was lower than that of the proposed method, partially due to the high ${ Se }$ achieved by the proposed method.
- $\bullet$ Although using the method $\#5$ to diagnose **NM** yielded a higher ${ Sp }$ than that of the proposed method, the ${ CA }$ was lower than that of the proposed method, partially due to the high ${ Se }$ achieved by the proposed method.
- $\bullet$ For other sounds, the classification accuracies achieved in the proposed method were all greater than those of the other methods listed in Table 7 .

Overall, the efficiency of the proposed method in diagnosing **MR**, **MS**, **ASD**, **NM**, **AS**, **AR** and **VSD** diseases was evaluated by comparison with the other efficient methods listed in Table 7.

**Table 6:** Efficient methods successfully used in diagnosing normal sounds from other common heart diseases.

Method	Year	Performance evaluation
$\sharp$ 1^46^	2021	The Fano-factor constrained tunable quality wavelet transform (TQWT) was the sensitivity and specificity of $86.32\%$ and $99.44\%$ respectively and overall score of $92.88\%$ to detect abnormal heart sounds.
$\sharp$ 2^47^	2021	This study proposed a heart sound classification method based on improved MFCC features and convolutional recurrent neural networks, which achieved classification accuracy of $98\%$ in the 2016 PhysioNeT/CinC Challenge database with dropout rate of 0.5.
$\sharp$ 3^48^	2020	A deep WaveNet model was proposed to classify five heart sound types and achieve high classification accuracies: $98.20\%$ for diagnosing Normal, $95.20\%$ for diagnosing MVP, $97.80\%$ for diagnosing MS, $96.10\%$ for diagnosing MR, $97.70\%$ for diagnosing AS.
$\sharp$ 4^8^	2018	The higher CA, achieved in this study, was $95.5\%$, $92.1\%$, $96.2\%$ and $99.0\%$ for diagnosing small ventricular septal defect (**VSD**), moderate **VSD**, large **VSD** and normal sounds, respectively.
$\sharp$ 5^49^	2017	A rule-based classification tree method proposed by this study achieved very high CA: $95.45\%$ for diagnosing **VSD**, $100\%$ for diagnosing normal, $100\%$ for diagnosing aortic stenosis and $95.45\%$ for diagnosing aortic insufficiency.
$\sharp$ 6^50^	2016	Artificial neural networks (ANNs) was reported to achieve the second-best score compared to the other methods in classifying the phonocardiogram recordings provided by the CinC Challenge.
$\sharp$ 7^51^	2016	Random forest, a meta-learning approach that uses multiple random decision trees as base learners and aggregates them to compute the final ensemble prediction, was successfully used in sound classification such as studies.

**Table 7:** Comparative analysis of eight different methods for the diagnosis of heart diseases summarized in Table 5.

Method	**MR**			**MS**			**ASD**			**NM**			**AS**			**AR**			**VSD**		
	${ Se }\%$	$CA (\%)$	$Sp (\%)$	${ Se }\%$	$CA (\%)$	$Sp (\%)$	${ Se }\%$	$CA (\%)$	$Sp (\%)$	${ Se }\%$	$CA (\%)$	$Sp (\%)$	${ Se }\%$	$CA (\%)$	$Sp (\%)$	${ Se }\%$	$CA (\%)$	$Sp (\%)$	${ Se }\%$	$CA (\%)$	$Sp (\%)$
$\sharp 1$	92.1	86.34	87.6	88.2	86.81	87.3	91.2	84.53	85.1	86.3	82.90	81.6	90.9	98.25	**99**.**1**	88.2	86.05	85.4	95.2	96.31	96.8
$\sharp 2$	90.6	89.93	88.3	88.8	90.32	91.3	83.9	86.12	87.21	95.9	98.3	97.7	96.3	96.1	96.02	87.1	86.31	85.9	90.6	89.3	88.1
$\sharp 3$	90.1	88.6	87.5	85.1	85.69	**99**.**2**	81.3	80.97	80.8	93.6	91.95	90.3	87.9	85.04	83.3	92.1	88.69	85.6	88.6	86.9	85.9
$\sharp 4$	90.1	87.34	86.7	83.2	86.81	87.3	90.2	83.13	82.5	85.7	81.90	80.6	91.3	90.40	90.3	87.2	84.04	83.4	96.2	97.66	97.8
$\sharp 5$	88.6	85.93	85.3	86.1	89.72	90.2	79.8	82.10	82.3	96.1	98.93	**99**.**9**	98.3	91.95	96.2	85.1	86.97	83.6	87.5	87.19	82.1
$\sharp 6$	88.1	87.6	87.8	83.1	89.36	90.2	80.3	81.67	81.8	92.6	91.63	91.3	83.9	86.04	86.3	90.1	84.69	83.6	87.6	86.03	85.9
$\sharp 7$	89.7	91.64	92.1	85.2	83.52	83.3	86.3	87.49	87.6	93.7	91.61	90.9	90.1	87.05	86.7	85.2	82.21	81.6	90.8	91.63	91.7
This	100	99.43	99.3	99.2	98.93	98.9	99.6	99.13	99.1	100	99.85	99.8	98.8	98.62	98.6	100	99.67	99.6	100	99.91	99.9

**Table 8:** The highest accuracies corresponding to the parameters set in every state-of-the-art method.

Method	Performance evaluation
$\sharp$ 1^46^	The highest classification accuracies were obtained by using the features described in Table 3 on page 28.
$\sharp$ 2^47^	The highest classification accuracies were obtained by using the 13-features extracted using MFCC algorithm.
$\sharp$ 3^48^	The highest classification accuracies were obtained by using the porposed WaveNet model consists of 6 residual blocks.
$\sharp$ 4^8^	The highest classification accuracies were obtained based on the rules described in a previous study^8^.
$\sharp$ 5^49^	The highest CA results were obtained based on the following rules.
	Rule 1: If the $8^{th}$ value of Lyapunov exponent $(\mathrm{LPE}_{8})$ $\ge 0.79$ and $(\mathrm{LPE}_{9})$ $\le 0.38$ then the heart is normal.
	Rule 2: If ${\text {LPE}}_{2}\le 0.17$ and $(\mathrm{LPE}_{8})$ $\le 0.79$, then the heart disease is **VSD**.
	Rule 3: If ${\text {LPE}}_{4}\ge 0.17$, ${\mathrm{LPE}}_{6}\le 0.39$, and ${\mathrm{LPE}}_{3}\le 0.56$, then the heart disease is **MR**.
	Rule 4: If ${\text {LPE}}_{5}\ge 0.17$, ${\mathrm{LPE}}_{4}\ge 0.67$, and ${\mathrm{LPE}}_{3}\ge 0.37$, then the heart disease is **MS**.
	Rule 5: If ${\text {LPE}}_{7}\ge 0.54$, ${\mathrm{LPE}}_{3}\ge 0.29$, and ${\mathrm{LPE}}_{5}\ge 0.49$, then the heart disease is **AR**.
	Rule 6: If ${\text {LPE}}_{8}\ge 0.39$, ${\mathrm{LPE}}_{5}\ge 0.72$, and ${\mathrm{LPE}}_{2}\le 0.68$, then the heart disease is **ASD**.
	Rule 7: If ${\text {LPE}}_{9}\ge 0.64$, ${\mathrm{LPE}}_{3} \ge 0.39$, and ${\mathrm{LPE}}_{7}\ge 0.21$, then the heart disease is **AS**.
	Rule 8: If none of these conditions are met, the **HS** is undefined.
$\sharp$ 6^50^	The most accurate results were obtained by the structure consisting of one input layer with 60 neurons, one hidden layer with 11 neurons and one output layer with five neurons.
$\sharp$ 7^51^	The most accurate results were obtained by setting the number of features at each node, the number of trees and the maximum depth of trees to 1, 108, and 36, respectively.
This method	The most accurate results were obtained for the diagnosis of **MR**, **MS**, **ASD**, **NM**, **AS**, **AR** and **VSD** at $Thv =0.4, 0.3,0.2, 0.2, 0.4, 0.1$ and 0.2, respectively.

## Conclusion

A novel intelligent system was proposed for diagnosing heart diseases with high $CA$. The innovation of this approach is primarily reflected in: 1) the automatic extraction of secondary envelope-based frequency features; 2) the automatic determination of **PCA**-based diagnostic features $\gamma _{_1}$, $\gamma _{_2}$ and $\gamma _{_3}$; and 3) the determination of adjustable confidence regions corresponding to the $\chi ^{2}$ distribution. The confidence regions are obtained by calculating the Mahalanobis distance, which is adjusted by the desired confidence level $\beta$, and the results were used as the classification criteria for diagnosing heart diseases. The procedure for the implementation of the intelligent system involved three stages. Stage 1 described the location and extraction of **STMHT**-based ${ CS }_{1}$ and ${ CS }_{2}$. In stage 2, in the frequency domain, a novel method was first proposed to generate the envelopes ${ CS _{1}}_{\mathrm{F_{E}}}$ and ${ CS _{2}}_{\mathrm{F_{E}}}$; then, based on the $Thv$ lines, $\mathrm{FF}$ was automatically extracted. Finally, based on **PCA**, the first three principal components, $\gamma _{1}$, $\gamma _{2}$ and $\gamma _{3}$, which expressed $86.7\%$ of the $\mathrm{FF}$ information, were determined and used as diagnostic features. In stage 3, the **GMM**-based objective function $f_{ et }({\mathbf{x }})$ with respect to the features ${\mathbf{x}}=[\gamma _{1}, \gamma _{2}, \gamma _{3}]$ and the parameters [$\pi _{k}$, $\mu _{k}$, $\Sigma _{k}$], where $k=1, 2, \ldots , K$, was generated. Then, the $\chi ^{2}$ distribution for component *k* was determined by calculating the Mahalanobis distance from ${\mathbf{x }}$ to the class mean $\mu _{_k}$ of component *k*, and the confidence region for component *k* was determined by adjusting the optimal confidence level $\beta _{k}$ and used as the criterion (denoted as ${\mathrm{MDC}}_{k}$) to diagnose a given **HS**. The performance evaluation was validated by sounds from online **HS** databases and clinical heart databases. The accuracy of the proposed method was compared to the accuracies of other well-known classifiers, and the highest classification accuracies of $99.43\%$, $98.93\%$, $99.13\%$, $99.85\%$, $98.62\%$, 99.67$\%$ and 99.91$\%$ in the detection of **MR**, **MS**, **ASD**, **NM**, **AS**, **AR** and **VSD** sounds were achieved by setting $\beta _{k}(k=1, 2, \ldots , 7)$ to 0.87,0.65,0.67,0.65,0.67,0.79 and 0.87, respectively. Therefore, this proposed intelligent diagnosis system provided an efficient way to diagnose seven types of heart diseases.

The advantages and limitations were summarized as follows:

$\bullet$ **Advantages:** ${\textcircled {1}}$ ${CS}_{1}$ and ${CS}_{2}$ were automatically extracted to reduce difficulty in segmenting each cardiac cycle into a sequence of four heart stages: ${ S }_{1}\longrightarrow$ *Systole period* $\longrightarrow { S }_{2}\longrightarrow$ *Diastole period*; ${\textcircled {2}}$ More features could be extended by setting even more threshold values for the unknown heart diseases, especially for the heart sound with the compound heart diseases; ${\textcircled {3}}$ Every classifier achieved in this study could be adjusted based on the desired $\beta$ for fitting incremental new features without being retrained via huge training features.

**Figure 10:**
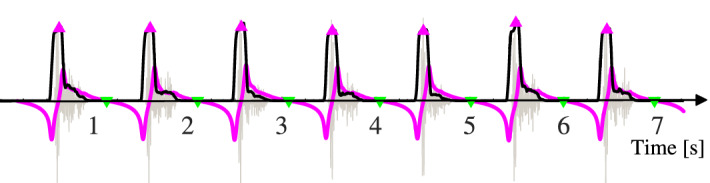
An example of a **AR** sound from database^26^.

$\bullet$ **Limitations:** ${\textcircled {1}}$ This methodology was impossible to diagnose the sounds when ${ CS }_{1}$ and ${ CS }_{2}$ cannot be segmented and extracted via the **STMHT** method for a given heart sound such as that plotted in Fig. 10; ${\textcircled {2}}$ The proposed classifier might not be satisfied with the compound heart diseases due to the distribution of features extracted from which can not fit a single Gaussian distribution.

## Future study

Future study focused on how to handle the sounds (such as some **AR** sounds) when ${ CS }_{1}$ and ${ CS }_{2}$ cannot be segmented and extracted via the **STMHT** method will be explored, and on how to build the classifier model for fitting the compound heart diseases will be further studied.

## Research statement

The study was conducted at Nanyang Institute of Technology and Nanyang First People’s Hospital, Henan, China from December 2017 to June 2021, and was approved by the ethics committee of Nanyang Institute of Technology and First People’s Hospital (Approval Number: V6.0). Informed consent was waived due to the retrospective design of the study. The study complies with the Declaration of Helsinki.

## References

[1] Tan, Z., Wang, W., Zong, R., Pan, J. & Yang, H. [classification of heart sound signals in congenital heart disease based on convolutional neural network]. *Sheng wu yi xue gong cheng xue za zhi = J. Biomed. Eng. Shengwu yixue gongchengxue zazhi* **36**, 728–736, 10.7507/1001-5515.201806031 (2019). 10.7507/1001-5515.201806031 PMC9935138 31631620

[2] Sun S, Wang H, Chang Z, Mao B, Liu Y (2019). On the Mahalanobis distance classification criterion for a ventricular septal defect diagnosis system. IEEE Sens. J..

[3] Omari T, Bereksi-Reguig F (2019). A new approach for blood pressure estimation based on phonocardiogram. Biomed. Eng. Lett..

[4] Notario, P. M. *et al.* Home-based telemedicine for children with medical complexity. *Telemed. e-Health* (2019). 10.1089/tmj.2018.0186 PMC6842894 30628860

[5] Coviello, J. S. *Auscultation Skills: Breath & Heart Sounds. -5th Ed.* (Lippincott Williams & Wilkins, 2013), 5 edn.

[6] Liu Q, Wu X, Ma X (2018). An automatic segmentation method for heart sounds. Biomed. Eng. Online.

[7] Messner E, Zöhrer M, Pernkopf F (2018). Heart sound segmentation: an event detection approach using deep recurrent neural networks. IEEE Trans. Biomed. Eng..

[8] Sun S, Wang H (2018). Principal component analysis-based features generation combined with ellipse models-based classification criterion for a ventricular septal defect diagnosis system. Aust. Phys. Eng. Sci. Med..

[9] Sun S (2015). An innovative intelligent system based on automatic diagnostic feature extraction for diagnosing heart diseases. Knowl. Based Syst..

[10] Shuping Sun ZJYF, Haibin W, Tao T (2014). Segmentation-based heart sound feature extraction combined with classifier models for a VSD diagnosis system. Exp. Syst. Appl..

[11] Choi S, Jiang Z (2010). Cardiac sound murmurs classification with autoregressive spectral analysis and multi-support vector machine technique. Comput. Biol. Med..

[12] Zhang J, Yin Z, Wang R (2017). Pattern classification of instantaneous cognitive task-load through GMM clustering, Laplacian eigenmap, and ensemble SVMS. IEEE/ACM Trans. Comput. Biol. Bioinf..

[13] Li Z, Xia Y, Ji Z, Zhang Y (2017). Brain voxel classification in magnetic resonance images using niche differential evolution based Bayesian inference of variational mixture of Gaussians. Neurocomputing.

[14] Ortiz-Rosario A, Adeli H, Buford JA (2017). MUSIC-Expected maximization gaussian mixture methodology for clustering and detection of task-related neuronal firing rates. Behav. Brain Res..

[15] Davari, A., Aptoula, E., Yanikoglu, B., Maier, A. & Riess, C. GMM-based synthetic samples for classification of hyperspectral images with limited training data. *IEEE Geosci. Remote Sens. Lett.* **15**, 942–946, 10.1109/LGRS.2018.2817361 (2018). arXiv:1712.04778.

[16] Simms, L. M. *et al.* Nuclear Inst . and Methods in Physics Research , A Pulse discrimination with a Gaussian mixture model on an FPGA. *Nucl. Inst. Methods Phys. Res. A* **900**, 1–7, 10.1016/j.nima.2018.05.039 (2018).

[17] Xue W, Jiang T (2018). An adaptive algorithm for target recognition using Gaussian mixture models. Meas. J. Int. Meas. Conf..

[18] Zhang S (2020). Segmentation of small ground glass opacity pulmonary nodules based on Markov random field energy and Bayesian probability difference. Biomed. Eng. Online.

[19] The World Medical Association Inc. DECLARATION OF HELSINKI Ethical Principles for Medical Research Involving Human Subjects Adopted. *WMA General Assembly, Somerset West, Republic of South Africa* 1–5 (2008).

[20] 3MCompany. 3m health care company. http://www.3M.com/Littmann (2019).

[21] Bernard Karnath, W. T. Auscultation of the heart. http://www.turner-white.com/pdf/hp_sep02_heart.pdf (2002).

[22] Ali MN, El-Dahshan E-SA, Yahia AH (2017). Denoising of heart sound signals using discrete wavelet transform. Circ. Syst. Signal Process..

[23] Máttar JA (1991). Systolic and diastolic time intervals in the critically ill patient. Crit. Care Med..

[24] Yeo TC (1998). Value of a doppler-derived index combining systolic and diastolic time intervals in predicting outcome in primary pulmonary hypertension. Am. J. Cardiol..

[25] Cui W, Roberson DA, Chen Z, Madronero LF, Cuneo BF (2008). Systolic and diastolic time intervals measured from doppler tissue imaging: Normal values and z-score tables, and effects of age, heart rate, and body surface area. J. Am. Soc. Echocardiogr..

[26] Implementation, C. M. Heart sounds databases-continuing medical implementation. http://www.cvtoolbox.com/index.html (2019).

[27] MacWalter, D. & MacWalter, G. Human heart sounds and murmurs. http://www.dundee.ac.uk/medther/Cardiology/hsmur.html (2019).

[28] Shuping Sun, H. W. A novel method-based secondary envelope extraction for heart sound analysis (2020).

[29] 3MDatabase. 50 heart and lung sounds library. http://solutions.3m.com/wps/portal/3M/en_EU/3M-Littmann-EMEA/stethoscope/littmann-learning-institute/heart-lung-sounds/heart-lung-sound-library/ (2019).

[30] Medical Sound Library. Auscultate - Learn Heart Sounds, Murmurs and Medical Auscultation (2019).

[31] AMBOSSMed. AMBOSS: Medical Knowledge Distilled (2019).

[32] Auscultation Sound. Heart Murmur-Mitral Regurgitation Auscultation Sound !!! Complete (2019).

[33] Sound, M. H. & Library, M. University of michigan heart sound and murmur library. http://www.med.umich.edu/lrc/psb/heartsounds/index.htm (2019).

[34] ThinkLabs. Thinklabs heart sound library. https://www.thinklabs.com/heart-sounds?hc_location=ufi (2019).

[35] Mohseni, S. S. Heart arrhythmias classification via a sequential classifier using neural network , principal component analysis and heart rate variation. *IEEE 8th International Conference on Intelligent Systems Heart* 715–722 (2016).

[36] Kavitha, R. & Kannan, E. An efficient framework for heart disease classification using feature extraction and feature selection technique in data mining. In: *Proceedings of the 1st International Conference on Emerging Trends in Engineering, Technology and Science, ICETETS 2016*10.1109/ICETETS.2016.7603000 (2016).

[37] Guo, H.-W. *et al.* Heart rate variability signal features for emotion recognition by using principal component analysis and support vectors machine. In: *Proceedings of the 2016 IEEE 16th International Conference on Bioinformatics and Bioengineering (BIBE)* 274–277, 10.1109/BIBE.2016.40 (2016).

[38] Motin, M. A. Principal component analysis : a novel approach for extracting respiratory rate and heart rate from photoplethysmographic signal.**22**, 766–774 (2018). 10.1109/JBHI.2017.2679108 28287994

[39] H. El-Saadawy, H. A. S., M. Tantawi & Tolba, M. F. Electrocardiogram (ecg) heart disease diagnosis using pnn, svm and softmax regression classifiers. In: *The Eighth International Conference on Intelligent Computing and Information Systems (ICICIS)* 106–110 (2017).

[40] Johnson, R. A. & Wichern, D. W. *Applied Multivariate Statistical Analysis (6th Edition)* (Pearson, 2007).

[41] Dempster AP, Laird NM, Rubin DB (1977). Maximum likelihood from incomplete data via the em algorithm. J. R. Stat. Soc. Ser. B (Methodol.).

[42] Pinto RC, Engel PM (2015). A fast incremental gaussian mixture model. PLoS ONE.

[43] Proïa F, Pernet A, Thouroude T, Michel G, Clotault J (2016). On the characterization of flowering curves using Gaussian mixture models. J. Theor. Biol..

[44] Mungai, P. K. Using Keystroke Dynamics in a Multi-level Architecture to Protect Online Examinations from Impersonation. In: *Proceedings of the 2017 IEEE 2nd International Conference on Big Data Analysis* 622–627 (2017).

[45] Aryafar, A., Mikaeil, R., Doulati Ardejani, F., Shaffiee Haghshenas, S. & Jafarpour, A. Application of non-linear regression and soft computing techniques for modeling process of pollutant adsorption from industrial wastewaters. *J. Min. Environ.* (2018).

[46] Sawant NK, Patidar S, Nesaragi N, Acharya UR (2021). Automated detection of abnormal heart sound signals using Fano-factor constrained tunable quality wavelet transform. Biocyber. Biomed. Eng..

[47] Deperlioglu O (2021). Heart sound classification with signal instant energy and stacked autoencoder network. Biomed. Signal Process. Control.

[48] Oh SL (2020). Classification of heart sound signals using a novel deep WaveNet model. Comput. Methods Prog. Biomed..

[49] Karar ME, El-Khafif SH, El-Brawany MA (2017). Automated diagnosis of heart sounds using rule-based classification tree. J. Med. Syst..

[50] Zabihi, M. *et al.* Heart sound anomaly and quality detection using ensemble of neural networks without segmentation. *Comput. Cardiol. Conf. (CinC)* (2016) 10.22489/CinC.2016.180-213.

[51] Witten, I. H., Frank, E. & Hall, M. *Data Mining: Practical Machine Learning Tools and Techniques * (Morgan Kaufmann, 2016), fourth edn. arXiv:1011.1669v3.

